# FOLFIRINOX Chemotherapy in Metastatic Pancreatic Cancer: A Systematic Review and Meta-Analysis of Retrospective and Phase II Studies

**DOI:** 10.3390/jcm7010007

**Published:** 2018-01-04

**Authors:** Stephane Thibodeau, Ioannis A. Voutsadakis

**Affiliations:** 1 Northern Ontario School of Medicine, Sudbury, ON P3E 2C6, Canada; sthibodeau@nosm.ca; 2 Division of Clinical Sciences, Northern Ontario School of Medicine, Sudbury, ON P3E 2C6, Canada; 3 Algoma District Cancer Program, Sault Area Hospital, Sault Ste. Marie, ON P6B 0A8, Canada

**Keywords:** FOLFIRINOX, chemotherapy, pancreatic cancer, efficacy outcomes, adverse effects

## Abstract

The introduction of the FOLFIRINOX regimen within the last decade marked the first progress in the clinical field of metastatic pancreatic cancer which had not seen any improvements in treatment availability for several years. In a phase III randomized clinical trial, FOLFIRINOX showed superior efficacy compared to the previous standard treatment of gemcitabine monotherapy. Nevertheless, it is unknown whether the superior results observed in this single phase III clinical trial can be translated more broadly to clinical practice. Our investigation sought to analyze all published evidence of the FOLFIRINOX regimen in series and phase II trials and compare it to the experience of the phase III study. Survival analysis revealed that FOLFIRINOX was associated with an Overall Survival of 10–11 months both in the trials and in off-trial settings, with response rates also similar in both settings. The adverse effect profile was consistent between the pooled phase II and off-trial experience and the FOLFIRINOX regimen arm observed in the randomized phase III trial.

## 1. Introduction

Metastatic pancreatic cancer remains one of the most lethal diseases with 5-year survival in the low single digits [1]. For several years since its introduction gemcitabine monotherapy was the standard palliative treatment for this disease. Gemcitabine was approved based on a combined benefit of symptom palliation and survival, though its survival prolongation benefit is modest at best. Frustratingly, attempts to improve on these mediocre benefits of gemcitabine had been met with little success and no new treatments were established for metastatic pancreatic cancer for several years. Against this background, significant enthusiasm was generated when a phase III randomized clinical trial comparing gemcitabine with the combination regimen FOLFIRINOX (consisting of the combination of 5-fluorouracil, leucovorin, irinotecan, and oxaliplatin) showed an unprecedented survival benefit [2]. FOLFIRINOX quickly became the new standard for patients with metastatic pancreatic cancer who could tolerate it. However, besides this randomized study, no other randomized phase III confirmatory evidence exists for the FOLFIRINOX regimen in terms of its efficacy outcomes or for its feasibility. During the last few years, though, a few phase II studies and other retrospective and prospective series have been published examining FOLFIRINOX in the setting of metastatic pancreatic cancer. The current report seeks to pool this non-randomized evidence to provide data on the efficacy and toxicity of FOLFIRINOX in metastatic pancreatic cancer beyond the single phase III study that led to its introduction.

## 2. Methods

A search in major databases Medline/PubMed and Embase was performed to identify publications on the FOLFIRINOX regimen for the treatment of metastatic pancreatic cancer. Search terms used included “FOLFIRINOX” and “metastatic pancreatic cancer”. Phase II studies or retrospective and prospective series of FOLFIRINOX in patients with metastatic pancreatic adenocarcinoma were considered for inclusion. Studies included were in the English language. Studies describing patients with localized disease were excluded even if non-resectable. In studies that included both metastatic and locally advanced non-resectable patients, patients with metastatic disease were included in the pooled analysis only if data specifically for metastatic patients could be extracted from the data provided in the publication. A manual search of references of retrieved articles for additional relevant publications was also performed.

Data from included studies describing the population treated as well as treatment efficacy and toxicity parameters were extracted and pooled. Characteristics of patients with metastatic pancreatic cancer treated with the FOLFIRINOX regimen that were extracted and tabularized for the analysis included age at diagnosis, ECOG performance status (PS), number and type of previous lines of treatment for metastatic disease and number and site of organs involved. Efficacy outcomes of interest included median Overall Survival (OS), median Progression-Free Survival (PFS), Response Rate (RR) and Disease Control Rate (DCR). Overall and grades 3 and 4 toxicity rates were additional outcomes of interest for this pooled analysis. 

A comparison of the pooled data was made with the FOLFIRINOX arm of the single published randomized phase III study of this regimen in metastatic pancreatic cancer to determine whether the populations of metastatic pancreatic cancer patients treated in the phase III trial, treated in phase II studies, and reported off-trial in various series were similar and whether the regimen had a similar efficacy and toxicity profile in phases II and III trials and off-trials.

Descriptive statistics were calculated for all patients’ characteristics of interest and outcome measures. Some series included in the analysis did not present data for every characteristic or outcome of interest, and thus, means and confidence intervals were calculated using as the denominator the total number of patients included in those series that had data of the given characteristic or outcome of interest available. The number of series from which each outcome of interest was derived was also determined and presented. Pooled outcomes rates were weighted according to the number of patients in each series. Heterogeneity among the studies was evaluated with the Cochran’s Q and *I*^2^ tests. A fixed or random effect model was used if heterogeneity was low or high respectively [3]. Calculations were performed in Excel (Microsoft Corp., Redmond, WA, USA) as previously described with some modifications, as needed [4].

## 3. Results

**Figure 1:**
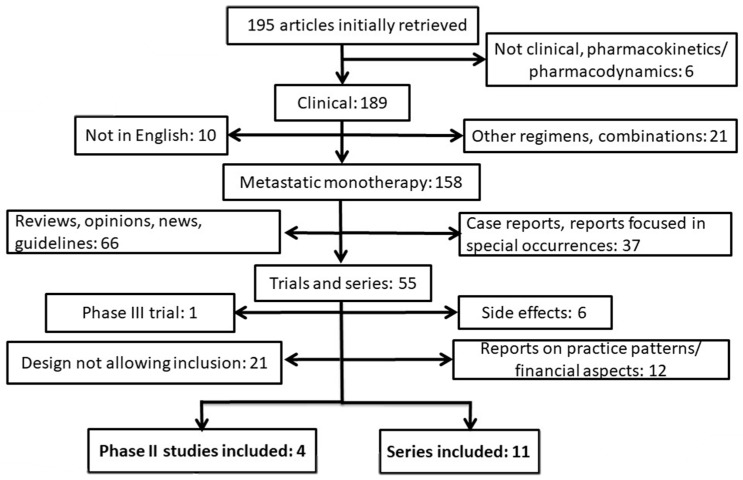
Diagram of the number of studies evaluated for these analyses and the reasons for exclusion. Eleven series and four phase II studies were included and pooled separately.

One hundred and ninety-five publications were initially retrieved (Figure 1). Six studies were excluded because they were preclinical or they dealt with the pharmacokinetics and pharmacodynamics of the drug combination. Additionally, thirty-one studies were excluded because there were not in English or were discussing other regimens or combinations. One hundred three further studies were excluded because they were reviews, opinions, news, guidelines and case reports or small series focusing on special clinical occurrences. Forty reports on financial and practice aspects of FOLFIRINOX, special adverse effects and reports with a design not allowing data extraction on efficacy and toxicity were also excluded (Figure 1). After these exclusions, eleven case series [5,6,7,8,9,10,11,12,13,14,15] and four phase II trials [16,17,18,19] remained and were included in the analyses of this report (Table 1 and Table 2). The eleven retained case series included 19 to 66 evaluable patients, were published between 2011 and 2016 and described a total of 354 patients (Table 1). All but one publication, which was from Asia [5], were from Europe and North America [6,7,8,9,10,11,12,13,14,15]. The majority of patients (51.6%) had a performance status of 0 while 36.1% had a PS of 1 and only 12.3% had a PS of 2 (Table 3). The primary tumor was located in the head of the pancreas in 57.3% of patients who had these data available and in the body or tail of the pancreas in 42.7%. Most patients had liver metastases, but a significant minority also had lymph node, lung or peritoneal metastases. The great majority of the patients with available data (83.9%) had FOLFIRINOX treatment as their first line of palliative chemotherapy (Table 3). The median number of cycles administered ranged from 4 to 16 in the four series in which this information was provided. Nine of the eleven series included in the analysis disclosed the dosing of their FOLFIRINOX regimen, which was the classic regimen proposed in the phase III trial, consisting of oxaliplatin 85 mg/m^2^, irinotecan 180 mg/m^2^, leucovorin 400 mg/m^2^, and 5-FU 400 mg/m^2^ as a bolus, followed by 2400 mg/m^2^ given as a continuous 46-hour infusion every 2 weeks. The two remaining series did not provide specific information on the version of the FOLFIRINOX regimen used. In the two series that provided data on FOLFIRINOX dose modifications, after the start of treatment, about two thirds of patients received a modified regimen. Among the four phase II trials identified and included in the meta-analysis, two [16,19] used the classic FOLFIRINOX regimen used in the phase III trial. The two others used a modified version, with irinotecan at 135 mg/m^2^ and 5-FU bolus at 300 mg/m^2^ [17], and irinotecan at 100 mg/m^2^ [18].

**Figure 2:**
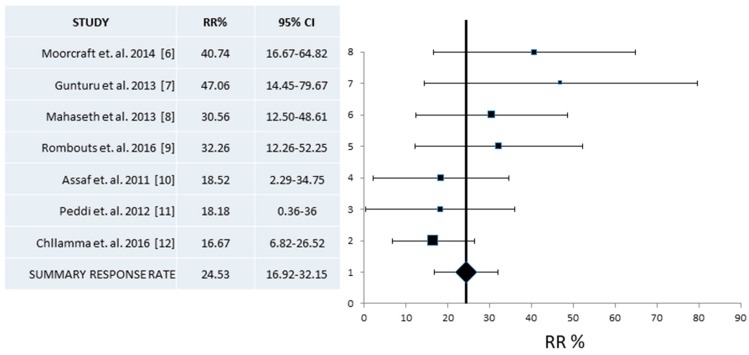
Pooled analysis of Response Rates (RR) and 95% Confidence Intervals (CI) from published series. Seven studies with a total of 229 patients that provided information for the RR were included and showed an overall RR of 24.5% (95% CI 16.9–32.1%).

A pooled analysis of the Response Rate (RR) was based on seven of the eleven off-trial studies that included the relevant data on this rate, referring to a total of 229 evaluable patients [6,7,8,9,10,11,12]. Heterogeneity among the seven studies was high with an *I*^2^ value of 23.4 (Cochran’s Q = 7.83, x^2^ *p* = 0.25). As a result, calculations were made under a random effect model. Overall pooled RR was 24.53% (95% CI 16.92–32.15%) (Figure 2).

**Figure 3:**
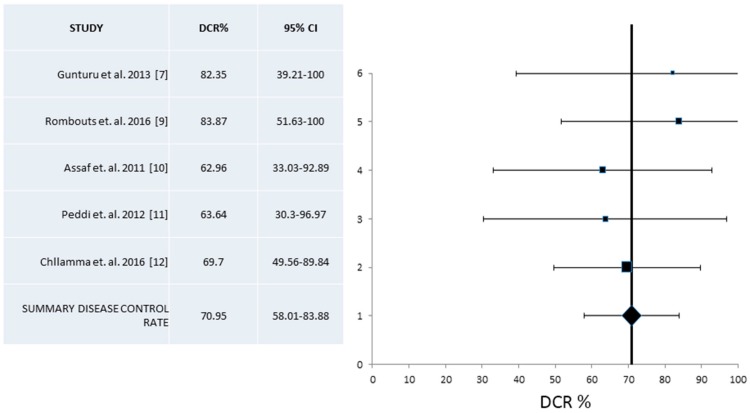
Pooled analysis of Disease Control Rates (DCR) and 95% Confidence Intervals (CI) from published series. Five studies with a total of 163 patients that provided information for the DCR were included. Overall, DCR was 70.95% (95% CI 58.0–83.9%).

The corresponding analysis for the Disease Control Rate (DCR) was based on five of the eleven studies with 163 evaluable patients [7,9,10,11,12]. A fixed effect model was used given that heterogeneity was low in this case (*I*^2^ = 0, Cochran’s Q = 1.35, x^2^ *p* = 0.85). Overall pooled DCR was 70.95% (95% CI 58.01–83.88%) (Figure 3).

**Figure 4:**
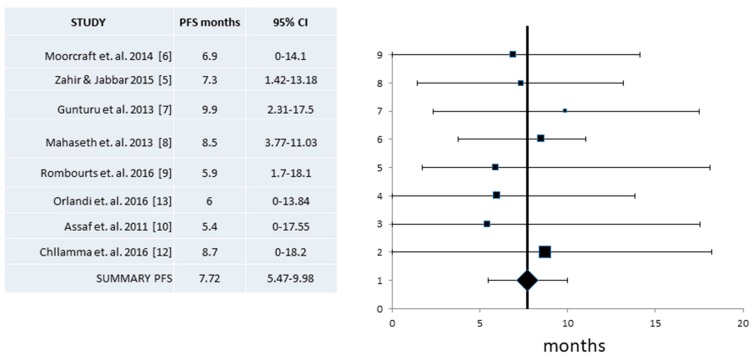
Pooled analysis of Progression-Free Survival (PFS) and 95% Confidence Intervals (CI) from published series. Eight studies with a total of 266 patients that provided information for the PFS were included in this analysis and revealed an overall PFS of 7.72 months (95% CI 5.47–9.98 months).

Progression-Free Survival (PFS) information was provided in eight of the eleven studies with a total of 266 patients [5,6,7,8,9,10,12,13]. Heterogeneity of the studies reporting PFS was low (*I*^2^ = 0, Cochran’s Q = 1.17, x^2^ *p* = 0.99) and a fixed effect model was employed. The pooled PFS was 7.72 months (95% CI 5.47–9.98 months) (Figure 4).

**Figure 5:**
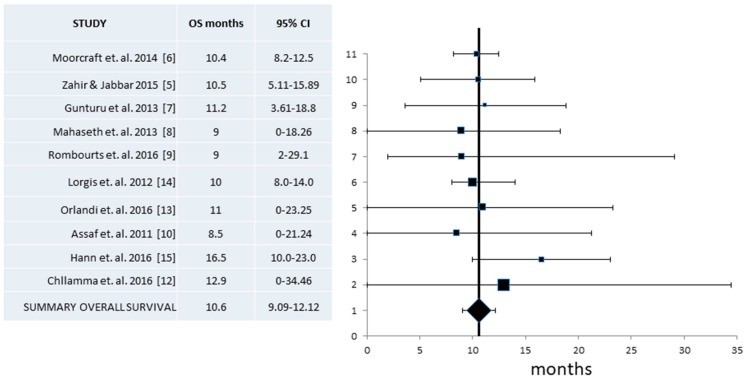
Pooled analysis of Overall Survival (OS) and 95% Confidence Intervals (CI) from published series. Ten studies with a total of 332 patients that provided information for the OS were included. Pooled OS derived was 10.6 months (95% CI 9.09–12.12 months).

Finally, data on Overall Survival (OS) were available from ten studies encompassing a total of 332 evaluable patients [5,6,7,8,9,10,12,13,14,15]. Heterogeneity was low in this case (*I*^2^ = 0, Cochran’s Q = 3.7, x^2^ *p* = 0.93), prompting the use of a fixed effect model. The resulting pooled OS was 10.6 months (CI 95% 9.09–12.12 months) (Figure 5).

**Figure 6:**
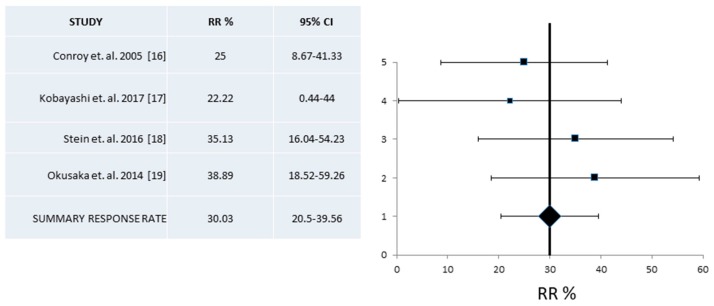
Pooled analysis of Response Rates (RR) and 95% Confidence Intervals (CI) from phase II trials. Four trials with a total of 127 patients that provided information for the RR were included and showed an overall RR of 30% (95% CI 20.5–39.6%).

**Figure 7:**
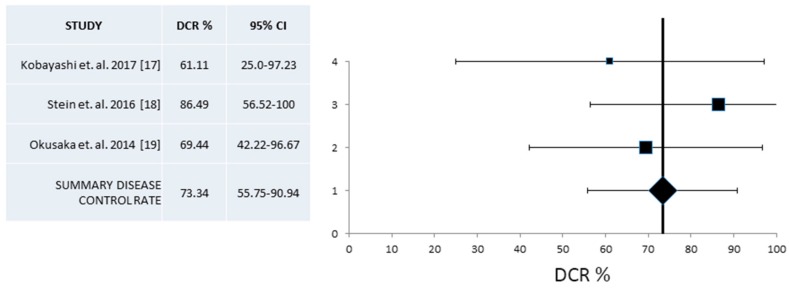
Pooled analysis of Disease Control Rates (DCR) and 95% Confidence Intervals (CI) from phase II trials. Three studies with a total of 91 patients that provided information for the DCR were included. Overall, DCR from these trials was 73.34% (95% CI 55.7–90.9%).

A pooled analysis of RR and DCR from phase II studies was based on four and three publications respectively [16,17,18,19]. In both cases heterogeneity was low with *I*^2^ = 0, Cochran’s Q = 1.85, x^2^ *p* = 0.6 and Cochran’s Q = 1.2, x^2^ *p* = 0.53, respectively. A fixed effect model was employed in both cases. Resulting pooled RR from phase II trials was 30% (95% CI 20.5–39.6%) (Figure 6) and DCR was 73.34% (95% CI 55.75–90.94%) (Figure 7).

**Figure 8:**
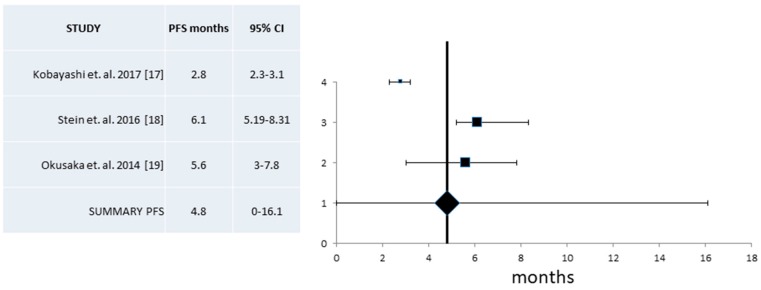
Pooled analysis of Progression-Free Survival (PFS) and 95% Confidence Intervals (CI) from phase II trials. Three trials with a total of 91 patients that provided information for the PFS were included in this analysis and revealed an overall PFS of 4.8 months (95% CI 0–16.1 months).

PFS from phase II studies resulted from the analysis of three trials which had a high heterogeneity, and thus a random effect model was used [17,18,19]. PFS was 4.8 months (95% CI 0–16.1 months) (Figure 8).

**Figure 9:**
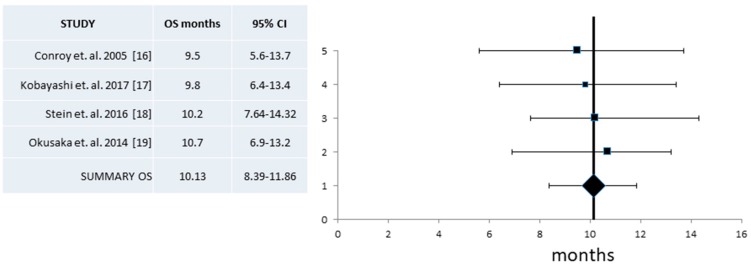
Pooled analysis of Overall Survival (OS) and 95% Confidence Intervals (CI) from phase II trials. Four trials with a total of 127 patients that provided information for the OS were included. Pooled OS derived was 10.13 months (95% CI 8.4–11.9 months).

Data on OS in phase II studies were pooled from all four retained publications and resulted in a mean of 10.13 months (95% CI 8.39–11.86 months) (Figure 9). Heterogeneity of the four studies with OS was low (*I*^2^ = 0, Cochran’s Q = 0.25, x^2^ *p* = 0.96) and thus a fixed effect model was utilized in this analysis.

The mean and median ages of patients in the pooled series and the phases II and III trials were similar overall, i.e., approximately 60 years old. Some series, though, had a somewhat lower mean age of included patients (Table 3). Regarding ECOG performance status (PS), the five series for which data were available included a small percentage (12.3%) of patients with a PS of 2, while both the phase II studies and the phase III trial had virtually only patients with PS of 0 or 1. The off-trial series also included a small percentage of patients (16.1%) with FOLFIRINOX as a later line of treatment, while the phase III trial had included only first line FOLFIRINOX treatment (Table 3).

A comparison of efficacy outcomes revealed that RR in the pooled studies was somewhat lower, in the range of 25%, than that of phases II and III trials, which were in the range of 30%, although the confidence intervals largely overlapped (Table 3). DCR were identical in series and trials at around 70%. Small differences in PFS were observed between series and phase II studies (7.7 versus 4.8 months, respectively), although again with largely overlapping confidence intervals. Additionally, the PFS of the FOLFIRINOX arm of the phase III trial was similar to that of the retrospective series at 6.4 months. OS was also similar in series and trials with medians of 10–11 months (Table 3).

The most common grades 3 and 4 adverse effects in the off-trial series with an incidence of above 10% included neutropenia (18.1%) and nausea/vomiting (10.2%). Despite that, the incidence of febrile neutropenia was only 4.3%, similar to the percentage in the phases II and III trials (Table 4). Other less common grades 3 and 4 adverse effects in the series with an incidence of 4% or more included diarrhea (6.3%), asthenia/fatigue (4.8%), thrombocytopenia (4.7%), neuropathy (4.1%), and mucositis (4%). The incidence of most of these grades 3 and 4 adverse effects was higher in the phase III trial, a fact that may at least partially be due to a more accurate recording prospectively as part of a clinical study. In addition, it is worth mentioning that in the pooled analysis of adverse effects in off-trial studies, several studies included both locally advanced and metastatic pancreatic cancer patients without presenting separately observed adverse effects. We have included those studies in the toxicity analysis regardless (Table 4), whereas in the efficacy analysis, these were excluded.

## 4. Discussion

The combination regimen FOLFIRINOX was introduced for the treatment of metastatic pancreatic adenocarcinoma after a multicenter randomized trial had proved its superiority compared to the previous standard of care consisting of gemcitabine monotherapy [2]. The introduction of this regimen ended several years of lack of new developments in the therapeutics of this aggressive malignancy and was met with expectations of further improvements. Soon thereafter, another regimen was introduced consisting of protein-bound paclitaxel and gemcitabine, increasing the available options for the treatment of this disease [20]. FOLFIRINOX, a regimen consisting of 5-fluorouracil, leucovorin, irinotecan, and oxaliplatin is now considered the first line standard of care therapy for metastatic pancreatic cancer patients that are deemed able to tolerate it. The change in practice that the introduction of the FOLFIRINOX regimen has had on the treatment of metastatic pancreatic adenocarcinoma is based on the results of the single randomized trial mentioned above [2]. This phase III multicenter randomized study performed by the French co-operative group showed an improvement in median PFS to 6.4 months in the FOLFIRINOX arm from 3.3 months in the control gemcitabine arm and in median OS from 6.8 months in the gemcitabine arm to 11.1 months in the FOLFIRINOX arm. These survival gains were consistent across patient subgroups and were associated with only moderate increases in grades 3 and 4 adverse effects such as neutropenia, febrile neutropenia, thrombocytopenia, diarrhea and sensory neuropathy.

Despite these clear survival improvements with FOLFIRINOX compared to the previous standard of gemcitabine monotherapy with only moderate increase in adverse effects, no confirmatory phase III evidence has been published so far. Thus, in this report, we sought to review and pool the available evidence of the regimen in metastatic pancreatic cancer from phase II studies and published series, to determine whether similar results as those achieved in the phase III randomized trial are observed in non-randomized and non-trial settings and in other populations. Both pooled phase II and off-trial series confirmed the OS observed in the FOLFIRINOX arm of the randomized phase III trial at about 10 to 11 months. The off-trial series showed a slightly better PFS at 7.7 months than the phase III trial, despite including a small number of patients with ECOG PS of 2 and also treated in later than first line of chemotherapy. The pooled estimate of PFS in phase II trials was actually lower at 4.8 months. RR and DCR in series and phase II trials demonstrated no significant differences from the phase III study. Regarding adverse effects, no unexpected grade 3 or 4 effects were observed in the pooled analyses and in fact rates of adverse effects were lower overall, although this may be related to incomplete capturing of this data in certain circumstances.

Some limitations of the current studies exist and include the heterogeneity of the pooled studies in quality and completeness of results reporting, necessitating exclusion of some studies from various components of the analysis. This may introduce bias and may reduce the power of the analyses by inclusion of fewer evaluable patients. In the same vein, most studies have included a mixture of metastatic and locally advanced patients and extraction of data for metastatic patients were not always feasible from the published articles.

## 5. Conclusions

Overall, our current pooled analyses of data on FOLFIRINOX in phase II studies and series are essentially consistent with the available phase III trial. This clinical information reinforces the value of FOLFIRINOX in metastatic pancreatic cancer for patients that have a preserved performance status and demonstrates that the regimen is a feasible treatment in clinical practice beyond the trial setting.

## Figures and Tables

**Table 1:** Off-trial series included in the pooled analysis of FOLFIRINOX in metastatic pancreatic cancer. The question-mark denotes that the study did not provide a Response Rate (RR) or Disease Control Rate (DCR).

Study [Reference]	Year of Publication	Country	Number of Patients	RR (%)	DCR (%)
Zahir & Jabbar [5]	2015	Pakistan	23	?	?
Moorcraft et al. [6]	2014	United Kingdom	27	40.7	?
Guruntu et al. [7]	2013	United States	19	47.1	82.4
Mahaseth et al. [8]	2013	United States	36	30.6	?
Rambouts et al. [9]	2016	The Netherlands	32	32.3	83.4
Assaf et al. [10]	2011	France	27	18.5	63.0
Peddi et al. [11]	2012	United States	22	18.2	63.6
Chllamma et al. [12]	2016	Canada	66	16.7	69.7
Orlandi et al. [13]	2016	Italy	36	?	?
Lorgis et al. [14]	2012	France	42	?	?
Hann et al. [15]	2016	Germany	24	?	?

**Table 2:** Phase II trials pooled in the current meta-analysis of FOLFIRINOX in metastatic pancreatic cancer. The question-mark denotes that the study did not provide the Disease Control Rate (DCR).

Study [Reference]	Year of Publication	Country	Number of Patients	RR (%)	DCR (%)
Conroy et. al. [16]	2005	France	36	25	?
Kobayashi et al. [17]	2017	Japan	18	22.2	61.1
Stein et. al. [18]	2016	United States	37	35.1	86.5
Okusaka et. al. [19]	2014	Japan	36	38.9	69.4

**Table 3:** Baseline characteristics and efficacy outcomes of patients included in the current pooled analysis of off-trial studies, the phase II studies, and the randomized phase III trial of FOLFIRINOX in metastatic pancreatic cancer. The third and fifth columns refer to the total number of patients and number of series on which the results depicted in the second and fourth columns, respectively, are based. Pts, patients.

Patients and Disease Variables	Pooled Series Pts (%)	Pooled Series: # of Pts/# of Series	Pooled Phase II Pts (%)	Pooled Phase II: # of Pts/# of Series	Phase III Trial
Study Size					
Evaluable Patients	368	368/11	126	126/4	171
Demographics					
Age—Mean	52–63	101/4			
Age—Median			61.5–63	91/3	61 (25–76)
Performance Status					
ECOG 0	80 (51.6)	155/5	51 (56.0)	91/3	64 (37.4)
ECOG 1	56 (36.1)	155/5	40 (44.0)	91/3	106 (62.0)
ECOG 2	19 (12.3)	155/5	0		1 (0.6)
Primary Location					
Head	82 (57.3)	143/5	29 (55.8)	52/3	67 (40.6)
Body/Tail	61 (42.7)	143/5	23 (44.2)	52/3	98 (59.4)
Metastatic Sites					
1	49 (46.7)	105/3			2 (Median)
≥2	56 (53.3)	105/3			1–6 (Range)
Metastatic Organs					
Liver	49 (71.0)	69/2	64 (70.3)	91/3	149 (87.1)
Lymph Nodes	29 (42.0)	69/2	39 (42.9)	91/3	49 (28.7)
Lung	21 (30.4)	69/2	13 (14.3)	91/3	33 (19.3)
Peritoneal	18 (26.1)	69/2	14 (15.4)	91/3	33 (19.3)
Prior Lines of Chemotherapy					
0	78 (83.9)	93/4			171
≥1	15 (16.1)	93/4			0
FOLFIRINOX Regimen					
Median Cycles	4–16	118/4	6–9	91/3	
Dose Reductions	34 (65.4)	55/2	69	70/2	
Efficacy					
RR% (95% CI)	24.5 (16.9–32.1)	229/7	30 (20.5–39.6)	127/4	31.6 (24.7–39.1)
DCR% (95% CI)	70.95 (58.0–83.9)	163/5	73.34 (55.7–90.9)	91/3	70.2 (62.7–76.9)
Median PFS (Mos) (95% CI)	7.72 (5.47–9.98)	266/8	4.8 (0–16.1)	91/3	6.4 (5.5–7.2)
Median OS (Mos) (95% CI)	10.6 (9.09–12.12)	332/10	10.13 (8.4–11.9)	127/4	11.1 (9.0–13.1)

**Table 4:** Toxicity of FOLFIRINOX in patients from the pooled analysis of retrospective studies, the phase II trials, and the randomized phase III trial. The third and fifth columns contain information on the total number of patients and number of series on which the results presented in the second and fourth columns, respectively, are based.

Toxicity	Pooled Series (%)	Pooled Series # of Pts/# of Series	Pooled Phase II (%)	Pooled Phase II: # of Pts/# of Series	Phase III Trial *N* = 171
Grade 3–4					
Neutropenia	18.1	514/10	18.3	174/4	45.7
Febrile Neutropenia	4.3	352/9	3.5	174/4	5.4
Anemia	2.6	371/7	4.8	174/4	7.8
Thrombocytopenia	4.7	537/11	4.0	174/4	9.1
Neuropathy	4.1	272/7	2.8	174/4	9.0
Nausea/Vomiting	10.2	457/10	5.5	174/4	14.5
Diarrhea	6.3	457/10	5.8	174/4	12.7
Asthenia/Fatigue	4.8	296/5			23.6
Mucositis	4.0	132/3			
Sepsis	2.5	69/2			
All Grades					
Neutropenia	31.2	156/5	24.0	54/2	
Febrile Neutropenia	4.0	190/3	4.5	54/2	
Anemia	35.7	171/3	24.0	54/2	
Thrombocytopenia	27.4	282/5	22.0	54/2	
Neuropathy	27.3	141/3	15.0	54/2	
Nausea/Vomiting	35.0	271/4	24.0	54/2	
Diarrhea	17.8	282/5	21.5	54/2	
Asthenia/Fatigue	23.4	282/5			
Mucositis	9.0	99/2			
